# The older adult surgical patient: a review of optimization and gaps in clinical practice

**DOI:** 10.1186/s13741-025-00593-x

**Published:** 2025-10-03

**Authors:** Cecilia Canales, Leslie Wann, Jeanna Blitz, Robert Whittington

**Affiliations:** 1 Department of Anesthesiology and Perioperative Medicine, UCLA David Geffen School of Medicine, Los Angeles, CA USA; 2 Donald and Barbara Zucker School of Medicine at Hofstra/Northwell, Hempstead, NY USA; 3 Department of Anesthesiology, Wake Forest University School of Medicine, Winston-Salem, NC USA

## Abstract

As the number of older adults undergoing surgery increases, the perioperative community must address the unique challenges of this vulnerable population. Age alone is not a sufficient indicator of risk and other factors like functional status, nutrition, and cognition also play a crucial role in determining a patient's vulnerability. Identifying high-risk patients requires targeted assessment to identify those who can most benefit from optimization. With an already strained perioperative workforce, integrating comprehensive geriatric programs**,** which rely on multidisciplinary teams to conduct frailty and geriatric assessments and to target prehabilitation and optimization**,** is a strategy to improve outcomes. By utilizing comprehensive geriatric programs that combine preoperative, intraoperative, and postoperative strategies to optimize care for older adults, the perioperative community can address the unique needs of high-risk older adults to reduce complications, mortality, and healthcare costs while improving the quality of life of these patients. In this review, we highlight strategies to optimize older adults undergoing surgery and identify significant gaps in practice that must also be addressed to improve the perioperative care of this often vulnerable patient population.

## Introduction

It is estimated that one in three surgical patients are now 65 years of age or older, and in the US alone, nearly 5 million older adults undergo surgery every year with this number expected to double by 2060 (Becher et al. 2023; The 2018 Ageing Report: Economic and Budgetary Projections for the EU Member States (2016–2070) 2018; Adeleke and Blitz 2021; Chow et al. 2012). Perioperative healthcare providers are increasingly recognizing the unique challenges of caring for older adults undergoing surgery and anesthesia. Identifying and addressing vulnerabilities in this population is becoming a critical priority. However, older adults represent a unique and heterogenous patient population. The concept of “high-risk” older adults extends beyond chronological age, encompassing factors such as functional status, mobility, social support, and nutritional, cognitive, and psychological health that influence resilience and recovery after surgery (Adeleke and Blitz 2021; McIsaac et al. 2020; Dent et al. 2019; Oresanya et al. 2014).

Older adults may be vulnerable in the perioperative period due to physiologic changes related to longevity (i.e. drug metabolism, elimination, frailty, etc.); however, most older adults survive surgery(Adeleke and Blitz 2021; McIsaac et al. 2017). Thus, as the proportion of older adult surgical patients increases, even marginal increases in age-related complications such as delirium, prolonged hospitalization, reduced days alive and out of hospital, and discharge to non-home location result in significant implications for healthcare expenditures and resource utilization (The 2018 Ageing Report: Economic and Budgetary Projections for the EU Member States (2016–2070) 2018; McGinn et al. 2023). Comprehensive programs or pathways that encompass a multidisciplinary approach to address the unique needs and challenges of high-risk older surgical patients are an important component of perioperative services and population health programs. By incorporating a range of healthcare professionals, including geriatricians, nurses, physical therapists, and social workers, comprehensive geriatric programs provide care that synergizes with the perioperative team’s efforts to optimize high-risk older adults, thus reducing overall costs, improving resource utilization, and improving overall patient outcomes. In this manuscript, we review the recommendations for perioperative optimization of high-risk patients, focusing on vulnerability rather than age as the determining factor, and we delineate knowledge gaps in clinical practice.

## The importance of identifying high-risk patients

Traditionally, perioperative healthcare providers are well-versed in assessing and identifying patients at high-risk for cardiac or respiratory complications and developing an anesthetic plan to mitigate these risks (Committee on Standards and Practice Parameters et al. 2012). In addition to cardiac and pulmonary risk factors, older adults may be at increased risk for perioperative complications that arise from the combination of physiological, psychological, and functional changes that occur with aging, many of which are often overlooked in the perioperative period (Vacas et al. 2022; Deiner et al. 2020). For example, frailty, a state of increased vulnerability, can result in changes in decreased physiological reserve to stressors, which can make it more challenging for older adults to cope with the stresses of surgery, anesthesia, and the postoperative recovery process (McIsaac et al. 2020; Hoogendijk et al. 2019). These changes can include slower metabolism and alterations in drug metabolism and elimination. Older adults have a higher prevalence of chronic health conditions, such as hypertension, diabetes and arrhythmias, which in turn affect renal, cardiac, and pulmonary function and can affect cognition (Vacas et al. 2022). These comorbidities and polypharmacy can increase the risk of perioperative complications and affect the body's ability to heal after surgery (Vacas et al. 2022). Furthermore, aging is associated with a decline in immune function and bone marrow suppression, which can lead to an increased susceptibility to infections after surgery, a slower wound healing process and inability to respond appropriately to acute blood loss (Vacas et al. 2022; Weyand and Goronzy 2016; Darden et al. 2020). Poor nutritional status is also common among older adults, which can impair wound healing and immune function (Martinez-Ortega et al. 2022). Furthermore, cognitive changes and slower thinking speed associated with aging can impact decision-making and understanding of perioperative instructions (Chow et al. 2012). Baseline cognitive impairment including dementia predisposes older adults to perioperative neurocognitive disorders including postoperative delirium and long-term cognitive decline, which can further complicate the recovery process(Kunicki et al. 2023; Whitlock et al. 2011). Lack of social support or other challenging living circumstances may impact the recovery trajectory after discharge and predispose the patient to additional risk including readmission and fewer days alive out of hospital (McDonald et al. 2018).

### Gaps in practice

For years, perioperative clinicians have been calling for routine preoperative assessment of older adults, focusing on cognitive changes, frailty, and nutrition (Chow et al. 2012; Oresanya et al. 2014; McIsaac et al. 2017; Berger et al. 2018; Hadley et al. 2017; Persico et al. 2018). Yet, over a decade later, major implementation gaps exist in the preoperative assessment and optimization of our older adults undergoing surgery with only 2.3% of respondents to a recent survey indicating they usually completed a frailty evaluation, and 0.7% indicating they usually conducted a preoperative comprehensive geriatric evaluations (Deiner et al. 2020). The aging population is placing increasing demands on healthcare systems, particularly by requiring more perioperative providers with expertise in older adult care (Becher et al. 2023). At the same time, there is a shortage of perioperative specialists, which contributes to gaps in implementing evidence-based strategies for optimizing older surgical patients (Becher et al. 2023). Without the identification and optimization of high-risk older adults, we can expect worse overall perioperative outcomes in this patient population (Etzioni et al. 2003; Vacas et al. 2021). Recognition of these factors is crucial for perioperative healthcare providers to tailor their care to the specific needs of older adults. There are numerous screening tools to aid in identifying high-risk older adults, but there remains significant variation in practice amongst providers of routine or widespread use of these tools (Table 1) (Deiner et al. 2020). For example, in a 2020 survey or anesthesiologists, those who worked in academic settings were more likely to conduct frailty screening (9.5% vs 3.4) in order to identify those who are high risk (Deiner et al. 2020). The goal is to rapidly identify those at high-risk for complications and then primarily focus on the time and resource-intensive interventions to optimize preoperative, intraoperative, and postoperative care to reduce complications and promote successful outcomes. Increasing implementation of screening tools specific to the vulnerabilities of older adults will allow providers to identify those patients and highest risk of adverse outcome and appropriately allocate optimization interventions that may be time- and resource-intensive to those that are most at risk.

**Table 1:**
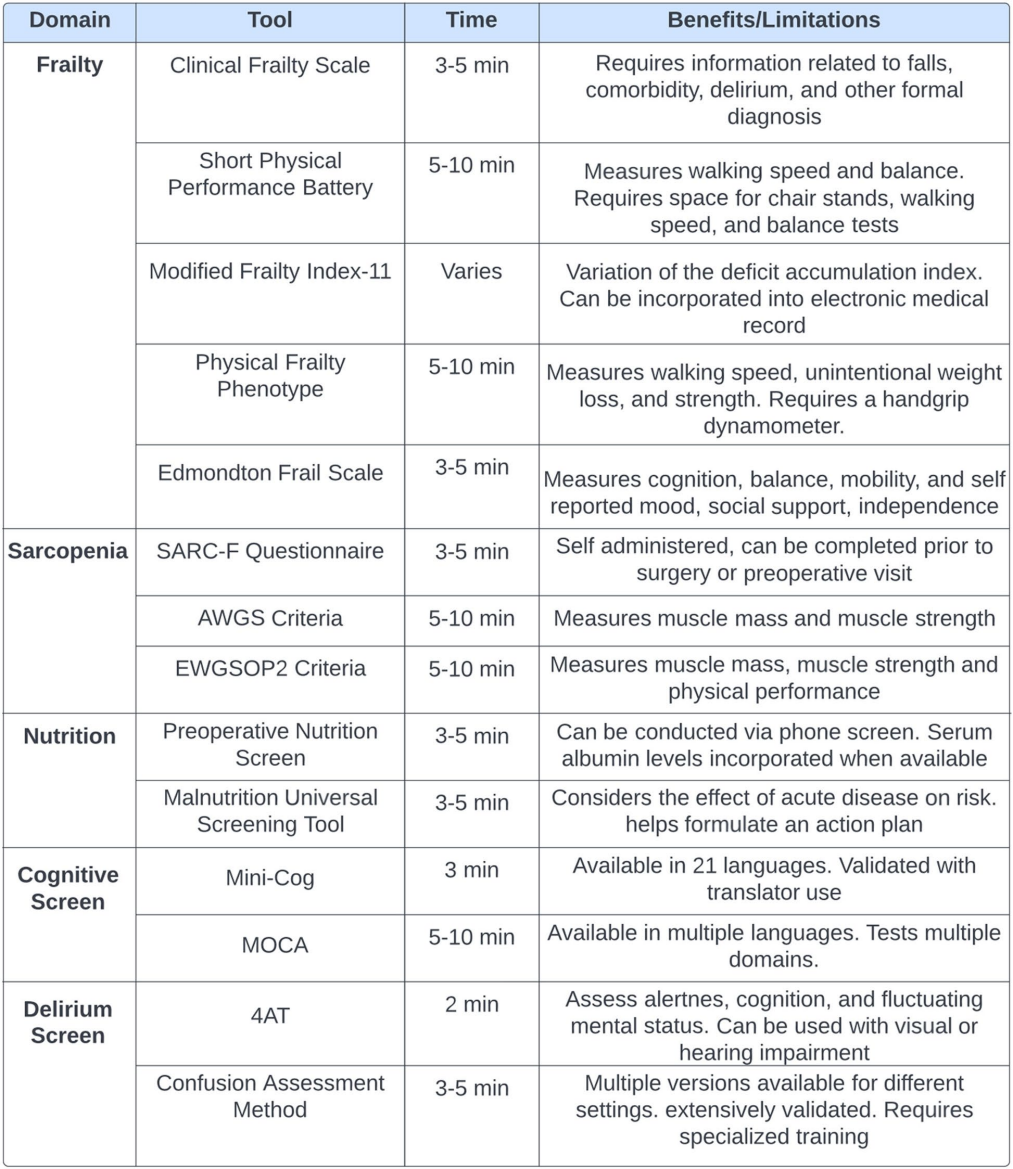
Screening tools for older adults

SARC-F=Strength, Assistance in walking, Rise from a chair, Climb stairs, and Falls; AWGS= Asian Working Group for Sarcopenia; EWGSOP2= European Working Group on Sarcopenia in Older People; MOCA= Montreal Cognitive Assessment; 4AT= Four A’s test (Alertness, Abbreviated Mental Test, Attention, Acute change or fluctuating)

Clinical frailty scale

https://www.ncbi.nlm.nih.gov/books/NBK559009/

Short physical performance battery

https://www.nia.nih.gov/research/labs/leps/short-physical-performance-battery-sppb

modified frailty index 11

https://pmc.ncbi.nlm.nih.gov/articles/PMC7477476/#:~:text=The%20modified%20frailty%20index,-The%20Canadian%20Study&text=The%20mFI%2D11%20was%20proven,%2D5%20and%20mFI%2D11.

Physical frailty phenotype

https://pmc.ncbi.nlm.nih.gov/articles/PMC8499264/

Edmondton frail scale

https://www.bgs.org.uk/sites/default/files/content/attachment/2018-07-05/efs.pdf

SARC-F questionnaire

https://pmc.ncbi.nlm.nih.gov/articles/PMC4799853/

AWGS criteria

https://pubmed.ncbi.nlm.nih.gov/32033882/

EWGSOP2 criteria

https://pmc.ncbi.nlm.nih.gov/articles/PMC6322506/

preoperative nutrition screen

https://pubmed.ncbi.nlm.nih.gov/34850403/

malnutrition universal screening tool

https://www.mdcalc.com/calc/10190/malnutrition-universal-screening-tool-must

Mini cog

https://mini-cog.com

MOCA

https://geriatrictoolkit.missouri.edu/cog/MoCA-8.3-English-Test-2018-04.pdf

4AT CAM

https://anesthesiaexperts.com/pacu-delirium-older-adults-aldrete-enough/

## Perioperative optimization strategies for high-risk older adults

### Pre-operative optimization of the geriatric patient

Prehabilitation refers to the process of enhancing a patient's physical and mental well-being before surgery with the aim of improving functional capacity, resilience, and optimizing overall health (Adeleke and Blitz 2021; Molenaar et al. 2023). There are several validated tools that can quickly identify patients who are at high risk for frailty and could therefore benefit from a more thorough evaluation such as a comprehensive geriatric assessment (CGA) (Mohanty et al. 2016; Pilotto et al. 2017). This therapeutic multidisciplinary diagnostic approach allows for perioperative optimization of the various aspects of frailty. There is well-established evidence that geriatrician review is associated with improved outcomes in planned surgeries such as hip fracture repair (Heghe et al. 2022). In addition to planned surgical intervention, there is now more recent evidence to support the benefit of CGA in emergency surgeries. Geriatrician review is associated with reduced mortality in both emergency laparotomy and trauma (Tian et al. 2023; Braude et al. 2022; Ibitoye et al. 2023; Oliver et al. 2018). CGA interventions can be tailored to patients’ specific needs such as improving functionality, nutrition, overall health status, cognition and psychosocial wellbeing. Prehabilitation and optimization prior to surgery have the capacity to improve postoperative outcomes (Adeleke and Blitz 2021; Shaw et al. 2023; Punnoose et al. 2023). While strategies including smoking cessation, chronic disease management of comorbidities such as diabetes, hypertension, obesity, and medication review are relatively common preoperatively, physical and cognitive resilience training, nutritional optimization, and focused collaborative care are relatively uncommon (Molenaar et al. 2023; Quan et al. 2019; Norris and Close 2020). Although there are mixed reports regarding the impact that prehabilitation studies have on perioperative outcomes, multiple studies of multipronged programs have demonstrated that tailored physical exercises can improve muscle strength, cardiovascular fitness, and overall mobility, which can help older adults build strength, thus facilitating recovery (Adeleke and Blitz 2021; McDonald et al. 2018; Molenaar et al. 2023; Punnoose et al. 2023; Waterland et al. 2021). Ensuring that older adults are well-nourished before surgery can also enhance their ability to recover (Martinez-Ortega et al. 2022). In community-dwelling adults, normal to slightly higher body mass index in frail older adults has been associated with improved all-cause mortality, suggesting that preoperative optimization of nutrition status can improve outcomes (Martinez-Ortega et al. 2022; Jayanama et al. 2022). Collaboration among the surgical team, primary care physicians, dietitians, and other healthcare professionals is crucial for designing an effective prehabilitation plan that addresses all aspects of the patient's health early enough to make a difference and improve outcomes without placing the burden of optimization solely on the perioperative team (McDonald et al. 2018; Molenaar et al. 2023).

### Gaps in practice

Despite the recognized importance, several gaps in practice remain in routinely implementing tools to identify and therapeutically optimizing of older adults. Specifically, some clinicians may not perform a thorough assessment of cognitive function, frailty, nutritional status, and functional status (Table 1). Furthermore, for those who do, there are often challenges in coordinating care or communication between different specialties(Ron et al. 2024). Time and resource requirements are often cited as barriers to implementation, therefore frailty screen or prehabilitation strategies, which include physical, nutritional, and psychological interventions, are not widely implemented. However, the additional costs associated with preoperative optimization can be offset by overall reduced healthcare expenditure in other areas such as hospital length of stay and complication rates. One study of patients undergoing prehabilitation prior to colectomy found an average savings of $21,946 per patient (Ron et al. 2024; Howard et al. 2019). Another study showed that even in a rural setting with limited resources, a multidisciplinary geriatric-focused surgical program could be successfully implemented (Ron et al. 2024).

Addressing these gaps requires a multifaceted approach involving education, policy changes, efficient allocation of resources and the implementation of standardized, evidence-based protocols to ensure comprehensive and consistent preoperative optimization for older adults.

### Intraoperative considerations for high-risk patients

Age-related alterations in physiology must be taken into consideration intraoperatively especially in high-risk patients. Intraoperatively, there should be a focus on keeping the patient euvolemic with adjusted hemodynamic goals to promote adequate perfusion pressure to vital organs including the heart and brain. The Beers list is a comprehensive list of potentially inappropriate medications that are usually best avoided in older adults under specific circumstances and a valuable resource for the care of older adult patients receiving anesthesia care (By the American Geriatrics Society Beers Criteria Update Expert P. American Geriatrics Society 2023 updated AGS Beers Criteria(R) for potentially inappropriate medication use in older adults 2023). Although more studies on Beers list medication use in the perioperative period are needed, anesthesia care providers should make every effort to minimize or, if possible, completely avoid the use of potentially inappropriate medications commonly administered intraoperatively (Table 2) (Curtis et al. 2020). Furthermore, age-related physiologic changes, such as reductions in the glomerular filtration rate and in the metabolic capacity of the liver, should be taken into consideration during anesthesia to ensure proper medication dosing.

**Table 2:** Potentially inappropriate drugs for older adults*

Perioperative Use	Drug	Uses in Perioperative Medicine	Alternatives
PONV	Dimenhydrinate Promethazine Scopolamine Diphenhydramine Metaclopromide Haloperidol Droperidol Dexamethasone Famotidine	Postoperative nausea and vomiting; sleep (sedative)	Ondansetron, Granisetron, Dolasetron, Aprepitant; Melatonin for sleep
Anxiolysis	Midazolam	Anxiolysis	Melatonin(Yousaf et al. 2010); Non-pharmacologic strategies (communication, patient education, music therapy)
Pain	Ketoralac High-dose Opioids Gabapentin	Postoperative pain	Acetaminophen, nerve blocks, patient-controlled epidural analgesia, carefully titrated opioids (Morphine, Oxycodone)
Shivering	Meperidine	Postoperative shivering	Dexmedetomidine (Abdel-Ghaffar et al. 2016)

^*^Adapted from: American Geriatrics Society 2023 updated AGS Beers Criteria(R) for potentially inappropriate medication use in older adults (By the American Geriatrics Society Beers Criteria Update Expert P. American Geriatrics Society 2023 updated AGS Beers Criteria(R) for potentially inappropriate medication use in older adults 2023)

Older adults lose muscle mass with age and have changes in metabolism, which may make them more susceptible to hypothermia (Chen et al. 2021). Therefore, it is also important to keep older adults normothermic with active warming throughout the perioperative period, as inadvertent hypothermia has been associated with worse perioperative outcomes (Sessler 2016). Other strategies for anesthetic management and monitoring remain controversial with various groups finding mixed and often contradictory results (Neuman et al. 2021; Wildes et al. 2019). Several studies have explored whether total intravenous anesthesia as compared to inhaled gas provide improved cognitive outcomes, while others have explored whether depth of anesthesia offers any improved results (Wildes et al. 2019; Li et al. 2022; Neuman et al. 2022). Although no consensus yet exists regarding type of anesthesia management for older adults, there is consensus that minimal alveolar concentration should be adjusted to age to reduce overexposure (Curtis et al. 2020; Aceto et al. 2020). There is also consensus that minimally invasive techniques and optimizing hemoglobin can improve outcomes (Curtis et al. 2020).

### Gaps in practice

Intraoperative management of high-risk older adults poses significant challenges, and several gaps in practice can impact outcomes. Most significantly is the lack of training and education for perioperative providers. There is limited training for anesthesiologists, surgeons, and perioperative staff on the unique needs and risks associated with high-risk older adults. There is a need for ongoing education and updates on best practices for managing older adults intraoperatively. It is also important to tailor guideline recommendations to each patient. The enhanced recovery after surgery (ERAS) protocol for example is an evidence-based pathway that promotes patient functioning and recovery. However, in older adults ERAS protocol could inadvertently introduce potentially inappropriate medications in patients at higher risk for postoperative delirium. Training and education can then help create individualized anesthetic plans that consider the specific physiological and pharmacological needs of high-risk older adults focused on pain management, delirium prevention, hemodynamic management and temperature regulation.

### Postoperative care and optimization strategies

Older adults, especially those with baseline cognitive impairment are at risk of postoperative delirium (Curtis et al. 2020). A single episode of postoperative delirium has consequences for short term outcomes including increased length of hospital stay, and long-term outcomes including increased risk of long-term cognitive decline (Kunicki et al. 2023; Curtis et al. 2020). Uncontrolled pain can predispose patients to postoperative delirium (Vlisides and Mashour 2021). Likewise, opioid analgesics can predispose to postoperative delirium (By the American Geriatrics Society Beers Criteria Update Expert P. American Geriatrics Society 2023 updated AGS Beers Criteria(R) for potentially inappropriate medication use in older adults 2023). Therefore, employing multimodal, opioid sparing techniques can reduce the risk of postoperative delirium (Curtis et al. 2020; Aceto et al. 2020). In a meta-analysis of over 3,000 patients, the use of postoperative regional techniques significantly reduced the relative risk of postoperative delirium when compared to systemic analgesia (Cooper et al. 2020). Other delirium prevention strategies include providing the patient with hearing aids, dentures, and glasses as early as possible (Thillainadesan et al. 2021). Furthermore, avoiding polypharmacy and potentially inappropriate medication administration can also improve perioperative outcomes (Burfeind et al. 2022). Implementing delirium management bundles, structured, multi-component interventions designed to prevent, detect, and manage delirium, improved overall outcomes (Inouye et al. 2000; Pun et al. 2019).

It is also important to screen for delirium in the postoperative period as often as possible with some groups screening every nursing shift given the fluctuating nature of this condition (Table 1) (Peig et al. 2021). Delirium can present as either hyperactive (e.g. agitation, restlessness, hallucination), hypoactive (e.g. lethargy, reduced motor activity) or mixed, and it is estimated that up to 50% of episodes, especially hypoactive presentations, go unrecognized and untreated leading to worse outcomes (Cooper et al. 2020; Robinson and Eiseman 2008). The confusion assessment method (CAM) and several specific versions of the tool, including the CAM-ICU, have been shown to have a high degree of sensitivity (94%) and specificity (89%) for detecting delirium (Partridge et al. 2021). Other important strategies for improving outcomes in high-risk older adults includes early focus on rehabilitation and functional recovery programs, as well as defining social work and family engagement in transitional care and discharge planning (Cooper et al. 2020).

### Gaps in practice

Several gaps in practice can impact postoperative outcomes in high-risk older adults. It can be difficult to balance effective pain management while avoiding overtreatment, particularly with deliriogenic medications. Delirium screening is inconsistently applied, leading to delayed diagnosis and treatment (Vacas et al. 2022; Deiner et al. 2020). Furthermore, there is a lack of implementation of evidence-based delirium prevention strategies, such as early mobilization, cognitive engagement, and sleep optimization. There is also limited education for patients and caregivers on postoperative care, warning signs of complications, and self-management strategies (Ragheb et al. 2023). Lastly, as many surgical procedures in older adults are now being performed on an ambulatory or short hospital stay basis, there is a need for cognitive function screening tools validated for use outside of the inpatient hospital setting.

## Perioperative geriatric program

Comprehensive geriatric programs encompass all the perioperative considerations of older adults that we have just highlighted. A comprehensive geriatric program plays a crucial role in determining the vulnerability of older adults undergoing surgical procedures. Several efforts have been established with the goal of reducing perioperative complications and mortality, enhancing functional outcomes and quality of life, improving patient satisfaction, reducing costs and improving resource utilization (Partridge et al. 2021; Cooper et al. 2022; Kamdar et al. 2020). The goal is to align multidisciplinary teams, provide comprehensive geriatric assessments with the goal of optimizing care, engaging patients and their families to align care goals, improve outcomes, and reduce costs. One example of a perioperative, interdisciplinary geriatric program called the Perioperative Optimization of Senior Health (POSH) found that their patients had significantly reduced length of stay, readmission rates, postoperative complications, and were more likely to be discharged home rather than to a facility compared to a matched historical group (McDonald et al. 2018). The major elements of this program include early identification of risk in the preoperative period, creation of an optimization plan specific to each patient, and postoperative interdisciplinary management by both surgery and geriatrics teams. Increased cost is often cited as a barrier to the implementation of such programs, but their interdisciplinary nature means that already existing resources can be used in a more collaborative and efficient way. A comprehensive geriatric assessment is a systematic evaluation of an older adult's medical, functional, cognitive, and psychosocial status. It involves a multidimensional approach to assessing the patient's overall health and well-being (Md Fadzil et al. 2022). Comprehensive geriatric assessments are important, as some vulnerabilities may not be immediately apparent. This holistic evaluation often performed by geriatricians, primary care physicians, and other trained providers is meant to identify hidden risks of falls, depression, frailty, cognitive impairment, and malnutrition (Welsh et al. 2014; Partridge et al. 2014). This allows for better risk stratification and the tailoring of healthcare plans and the facilitation of informed and shared decision making. Hence, families and healthcare providers can have realistic discussions about the risks and benefits of surgical intervention. By identifying vulnerabilities early, healthcare teams can implement targeted interventions before, during, and after surgery (Table 3). This proactive approach can lead to better and enhanced discharge planning helping healthcare teams arrange for appropriate post-surgical care including home support, rehabilitation services, and follow-up appointments. By considering the multidimensional aspects of health and tailoring interventions accordingly, healthcare teams can optimize surgical outcomes and improve the overall quality of care for older adults (Partridge et al. 2021, 2014).

**Table 3:** Preoperative optimization of the geriatric patient

Recommendations	Benefits	Evidence	Implementation Strategies	Alternatives
Screen for frailty	Identify and possibly intervene on patients who are at higher risk of postoperative complications, poor outcomes, prolonged hospital stay, hospital readmissions, increased 30-day and long-term mortality (Cappe et al. 2023)	In a meta-analysis of 9153 surgical patients, implementation of a frailty screening initiative was associated with reduced mortality (Hall et al. 2017)	Screen for frailty with a quick, validated tool during routine preoperative assessment At risk patients should be further assessed in the various domains of frailty (nutrition, physical health, mental health, social support, cognition)	If it is not possible to complete a frailty screening prior to surgery, patients should optimize their risk factors such as tobacco consumption, diabetes and cardiovascular disease All patients should be referred to an adapted rehabilitation program postoperatively to address the specific needs of a frail patient
Assess nutritional status	Optimizing a patient’s nutritional status prior to surgery can increase functional capacity, improve physical strength and optimize recovery postoperatively (Borloni et al. 2019)	In a study of abdominal surgery patients, preoperative nutritional support was found to decrease both hospital length of stay and complication rate in patients at nutritional risk (Jie et al. 2012)	Assess nutritional risk with a short screening tool High risk patients should be referred to a nutritionist to address deficits and determine the need for preoperative nutritional support	If referral to nutritionist or social work is not possible, standardized education should be given to all patients encouraging protein intake along with any specific deficiencies
Engage in prehabilitation	Prehabilitation has the potential to improve a patient’s preoperative condition and surgical outcome (Molenaar et al. 2023)	In a randomized trial comparing prehab to usual care in major abdominal surgery, prehab significantly reduced rates of postoperative complications and led to cost savings (Howard et al. 2019) In a meta-analysis of 700 older adults undergoing abdominal surgery, multi-modal prehab was found to increase perioperative functional capacity (Pang et al. 2022) Cognitive prehab has been shown to reduce the risk of post-operative delirium (Humeidan et al. 2021)	During standard preoperative evaluation, the physician should determine which elements of prehab would best suit each patient Prehab regimens that have been studied vary widely. A multimodal approach including exercise therapy, nutritional intervention, psychosocial intervention and medical assessment should address several needs of the geriatric patient	If supervised exercise programs and nutritionist-guided interventions are not possible, all patients should be encouraged to stay physically active, optimize their nutrition, quit/limit smoking and alcohol Physicians should medically optimize patient risk factors such as diabetes and cardiovascular disease

### Gaps in practice

There is inconsistent use and implementation of comprehensive geriatric assessments (CGA) to identify and address the multifaceted needs of older adults with insufficient involvement of a full multidisciplinary team, including geriatricians, anesthesiologists, surgeons, nurses, physiotherapists, and social workers. Challenges in effective communication and coordination among team members, often lead to fragmented care (Sum et al. 2022).

Addressing these gaps requires a concerted institutional effort to develop standardized assessment tools, foster multidisciplinary collaboration, implement comprehensive prehabilitation and rehabilitation programs, and enhance education and training for healthcare providers. Additionally, systemic changes in resource allocation, policy support, and the integration of technology are essential to optimize the perioperative care of older adults.

## Future directions and challenges

Implementing geriatric perioperative programs can be challenging due to various barriers that need to be addressed to ensure their effectiveness. First, despite recommendations being in place for over a decade, healthcare professionals may not be fully aware of the specific needs and vulnerabilities of older adults in the perioperative period, leading to inadequate or inappropriate planning (Deiner et al. 2020). Furthermore, there is often resistance to change from healthcare systems and providers to adopting new protocols or programs, especially if there is a concern for time, financial, or resource constraints. Addressing these perceived barriers will require a collaborative effort among healthcare professionals, administrators, policymakers, and patients. Developing comprehensive strategies that include education, training, resource allocation, and effective communication can help overcome these challenges and lead to successful implementation of geriatric perioperative programs for high-risk older adults (Russell et al. 2016).

Another significant challenge in the implementation of a geriatric perioperative program is a lack of consensus and standardization amongst recommendations. The ASA Committee on Geriatric Anesthesia and Perioperative Brain Health Initiative was formed with the goal of characterizing current practices in the perioperative care of older adults. They found that fewer than 10% of older adults were screened for frailty, pre-existing cognitive impairment, post-operative delirium, or received preoperative evaluation by a geriatrician (Deiner et al. 2020). When asked which initiatives should be prioritized to improve perioperative care of older adults, most respondents indicated specialty-specific practice guidelines written by the ASA (Deiner et al. 2020). Frailty screening, for example, is included in several perioperative guidelines, but many questions remain for healthcare providers. There are numerous frailty screening tools, and even more *definitions* of frailty, and physicians are left with the decision of which tool and definition is best suited for the older surgical patient. Future work should be done to identify which tools are best tailored to the older surgical population in order to maximize utility, improve provider adherence and patient outcomes.

There are opportunities to integrate technology and telemedicine into geriatric perioperative programs that can enhance care delivery, improve patient outcomes, and increase accessibility for older adults. For example, telemedicine platforms have already been adopted to conduct preoperative assessments and screenings before surgery (Cooper et al. 2022; Kamdar et al. 2020). Older adults can connect with healthcare providers virtually to discuss medical history, medications, nutrition, and other relevant information. Tele-rehabilitation programs guide older adults through exercise routines, functional mobility training, and activities of daily living exercises (Md Fadzil et al. 2022). Utilization of remote monitoring tools can help track postoperative progress and detect any early signs of complications (Md Fadzil et al. 2022). This can be particularly helpful for detecting issues such as postoperative delirium. However, it is important to ensure that technology integration is user-friendly, accessible, validated, and addresses the unique needs of older adults.

## Conclusion

By tailoring assessments and care plans targeting specific vulnerabilities of high-risk older adults, health systems and healthcare providers can reduce perioperative complications, mortality rates, and hospital readmissions. Furthermore, the optimization strategies employed by geriatric programs, such as nutritional support, cognitive assessment, medication review, and prehabilitation, contribute to improved functional outcomes as well as enhanced quality of life. Further attention to the specific gaps in practice identified in this review is warranted.

## Data Availability

No datasets were generated or analysed during the current study.
